# A Systematic Review of Immune Cell Roles in Breast Cancer Immunotherapy

**DOI:** 10.1002/cnr2.70217

**Published:** 2025-05-12

**Authors:** Rui Li, Wei Lv, Dong Liang Wang, Na Chen

**Affiliations:** ^1^ Shandong Provincial Hospital, Affiliated to Shandong First Medical University Jinan Shandong China; ^2^ Department of General Surgery Shandong Provincial Hospital Jinan Shandong China; ^3^ Urology, Shandong University Jinan Shandong China; ^4^ Department of Internal Medicine Shandong Provincial Hospital Jinan Shandong China

**Keywords:** breast cancer, immune cells, immunotherapy, tumor microenvironment, vaccines

## Abstract

**Background:**

Breast cancer (BC) is the most prevalent malignancy among women and is associated with high mortality and significant clinical challenges. Although conventional treatments such as surgery, chemotherapy, and radiotherapy have significantly improved patient survival, their efficacy remains limited by severe side effects and treatment resistance. In recent years, advances in immunotherapy have underscored the pivotal role of immune cells in treating BC.

**Recent Findings:**

This systematic review summarizes the current knowledge on the roles of immune cells within the BC tumor microenvironment (TME), including their phenotypes, functions, and implications for immunotherapy. Following PRISMA guidelines, 71 studies published between 2010 and 2024 were analyzed. The results indicate that immune cell populations—such as tumor‐associated macrophages (TAMs), tumor‐infiltrating lymphocytes (TILs), natural killer (NK) cells, dendritic cells (DCs), and myeloid‐derived suppressor cells (MDSCs)—are integral to tumor progression and therapeutic response. However, their functional heterogeneity and plasticity remain key obstacles to the development of effective and personalized immunotherapeutic strategies.

**Conclusion:**

Further research is needed to clarify the mechanisms governing immune cell behavior within the BC TME and to advance precision immunotherapy. Such insights will lay the foundation for individualized treatment approaches, ultimately improving patient outcomes and quality of life (QoL).

**Abbreviations**
ADCC: antibody‐dependent cellular cytotoxicity
BC: breast cancer
BCCL: breast cancer cell line
Bregs: regulatory B cells
CAFs: cancer‐associated fibroblasts
CAR‐NK/T: chimeric antigen receptor‐engineered NK/T cells
cDCs: conventional DCs
CTLs: cytotoxic CD8^+^ T lymphocytes
DCs: dendritic cells
ECM: extracellular matrix
FcγR: Fc gamma receptors
GEM‐CXB NP: gemcitabine‐curcumin‐loaded nanoparticles
GVHD: graft‐versus‐host disease
Hh: Hedgehog signaling pathway
ICIs: immune checkpoint inhibitors
ILCs: innate lymphoid cells
M1: pro‐inflammatory macrophages 1
M2: anti‐inflammatory macrophages 2
MDSCs: myeloid‐derived suppressor cells
MHC: major histocompatibility complex
mIHC: multiplex immunohistochemical staining
NETs: neutrophil extracellular traps
NK: natural killer
PCs: plasma cells
pDCs: plasmacytoid DCs
QoL: quality of life
SPP1: secreted phosphoprotein‐1
SRC‐3: steroid receptor coactivator‐3
TAMs: tumor‐associated macrophages
TANs: tumor‐associated neutrophils
tDLNs: tumor‐draining lymph nodes
Teff: effector T cells
Th17 Cells: T helper 17 cells
TILs: tumor‐infiltrating lymphocytes
TME: tumor microenvironment
TNBC: triple‐negative breast cancer
Tregs: regulatory T cells

## 1 Introduction

Breast cancer (BC) is the most prevalent malignancy among women worldwide, consistently demonstrating high morbidity and mortality rates. It has become the second leading cause of cancer‐related deaths in women, with an estimated 250 000 new cases anticipated over the next 25 years [1]. Despite substantial advancements in diagnosis and treatment modalities, the etiology of BC remains complex and multifactorial, involving intricate interactions between environmental, hormonal, genetic, and lifestyle factors [2]. Genetic mutations account for only approximately 5%–10% of BC cases, whereas modifiable risk factors contribute to approximately 20%–30% of occurrences [3]. Conventional treatments such as surgery, chemotherapy, and radiotherapy have significantly improved patient outcomes; however, postoperative recurrence and drug resistance remain significant clinical challenges [4].

Recent developments in immunotherapy have introduced promising strategies for BC treatment, mainly through the modulation of immune cell functions within the tumor microenvironment (TME). Tumors evade immune surveillance to facilitate their growth and remodel the TME to suppress immune cell activity [5]. Accumulating evidence indicates that immune cells in normal or tumor tissues play pivotal roles, directly influencing tumor progression and patient prognosis [6, 7]. Innate immune cells—including natural killer (NK) cells, macrophages, and dendritic cells (DCs)—are critical for early tumor recognition. In contrast, adaptive immune cells, such as T and B lymphocytes, mediate specific anti‐tumor responses and establish immunological memory [8, 9]. Nevertheless, our understanding of tumor immune evasion mechanisms remains limited, constraining further advances in immunotherapy.

**FIGURE 1:**
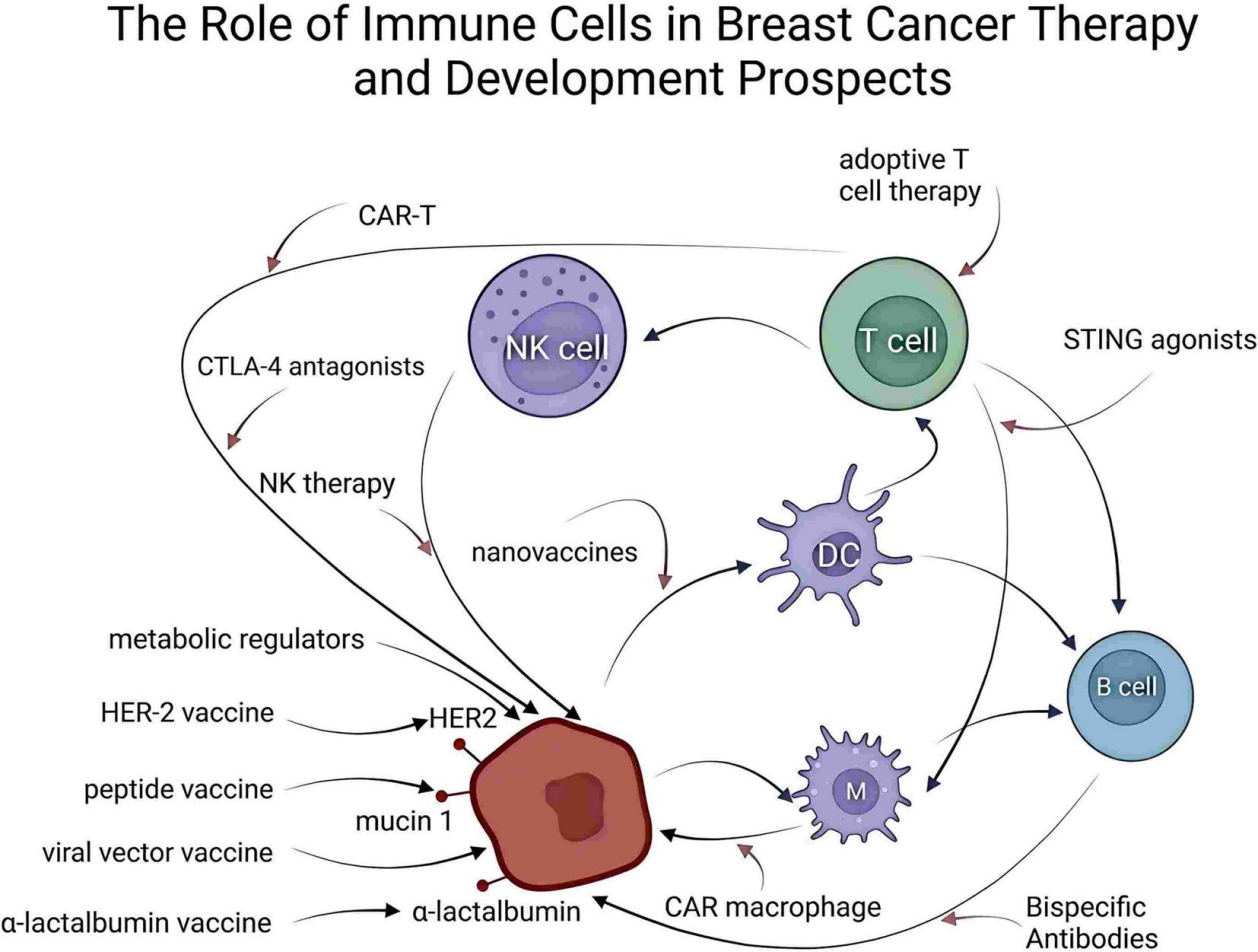
Graphical abstract illustrating the role and future prospects of immune cells in breast cancer therapy. This schematic summarizes the complex interplay among breast cancer (BC) cells (red), tumor‐associated antigens (including HER2, mucin 1, and α‐lactalbumin), and various immune cells such as dendritic cells (DCs), macrophages, T cells, B cells, and natural killer (NK) cells. The therapeutic strategies depicted include antigen‐targeted vaccines (HER‐2 vaccine, peptide vaccine, viral vector vaccine, α‐lactalbumin vaccine, and nano vaccines), cellular immunotherapies (CAR‐T cells, CAR‐macrophages, NK cell therapy, and adoptive T cell therapy), immune checkpoint modulation (CTLA‐4 antagonists and STING agonists), metabolic regulators, and innovative bispecific antibodies. Collectively, these approaches enhance antitumor immunity by activating effector immune cells, reversing tumor‐induced immunosuppression, and remodeling the tumor microenvironment (TME), thereby underscoring the potential for personalized and combination therapies in BC.

Immune checkpoint inhibitors (ICIs) targeting PD‐1/PD‐L1 pathways have demonstrated breakthroughs in melanoma and lung cancer treatments and have also exhibited promising efficacy and tolerability in triple‐negative BC (TNBC) [10]. However, the complexity of the TME and the heterogeneity of immune cell functions continue to pose significant obstacles to therapeutic success. A deeper understanding of the specific roles and dynamics of various immune cell subsets within the TME is essential to enhance existing immunotherapies and facilitate personalized treatment strategies. This review systematically examines recent advances in the BC immune microenvironment, focusing on key immune populations, including tumor‐infiltrating lymphocytes (TILs), NK cells, and DCs, as well as emerging immune mechanisms. The aim is to highlight current research gaps and provide theoretical and practical guidance for future clinical decisions and therapeutic innovations in BC precision immunotherapy (Figure 1).

## 2 Methods

### 2.1 Study Design and Guidelines

This systematic review used PRISMA guidelines (Supporting Information) to ensure transparency and methodological rigor.

### 2.2 Literature Search Strategy

Literature published between 2010 and 2024 was retrieved from PubMed, reflecting the rapid developments in BC immunology over the past decade. The following search strategy was employed:

(“Breast Neoplasms”[Mesh] OR “breast cancer” OR “mammary carcinoma”) AND (“Tumor Microenvironment”[Mesh] OR “immune microenvironment” OR “immune infiltration”) AND (“Immunotherapy”[Mesh] OR “immune cells” OR “immune response”) AND (“2010/01/01”[PDAT]: “2024/12/31”[PDAT]).

Initially, 2601 records were identified, with no duplicates removed.

### 2.3 Inclusion and Exclusion Criteria

Inclusion criteria were studies involving (1) BC patients, (2) immune microenvironment‐related cells and mechanisms, and (3) clinical or animal models. Exclusion criteria comprised: (1) non‐original studies (reviews, editorials), (2) unclear descriptions of immune cell functions, and (3) non‐English literature.

### 2.4 Study Selection

**FIGURE 2:**
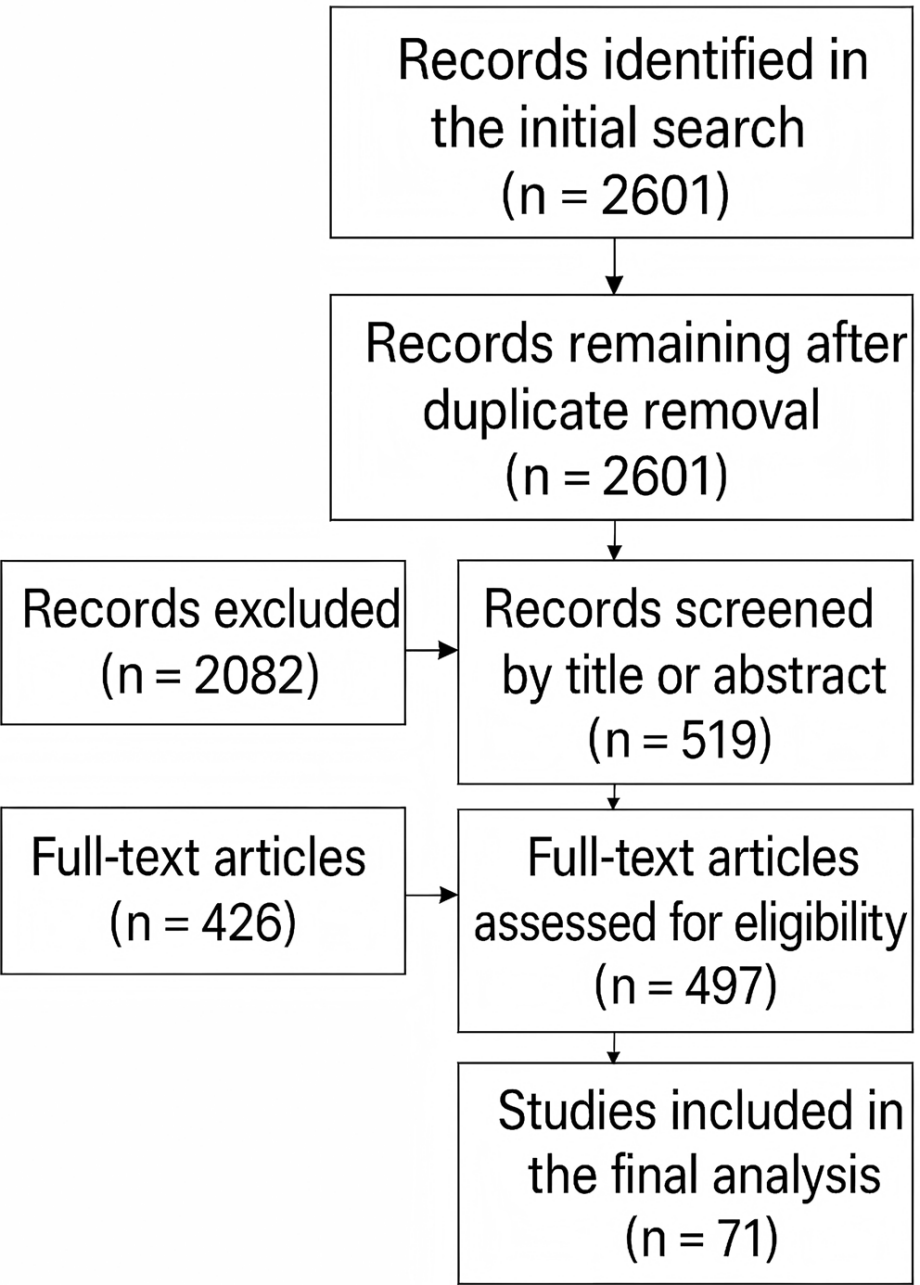
Literature selection flowchart based on the PRISMA 2020 Checklist. A total of 2601 records were initially identified through database searches, with the same number remaining after duplicate removal. Following a preliminary screening of titles and abstracts, 2082 records were excluded, leaving 519 records for full‐text review. After detailed evaluation, 426 records that did not meet the inclusion criteria were excluded, resulting in the final inclusion of 71 studies for the review. Detailed selection criteria and processes are provided in Supporting Information.

Based on the criteria above, 2082 records were excluded through title and abstract screening, leaving 497 for full‐text review. Twenty‐six articles were excluded due to inaccessible full text. A further 426 studies did not meet eligibility criteria upon detailed assessment, resulting in the inclusion of 71 articles for analysis. Figure 2 illustrates the detailed screening process.

### 2.5 Data Extraction and Processing

All variables were meticulously defined and standardized, specifying units, data sources, and extraction methods for consistency.

## 3 Overview of the BC Immune Microenvironment

### 3.1 Key Immune Cells and Mechanisms (Figure 3)

**FIGURE 3:**
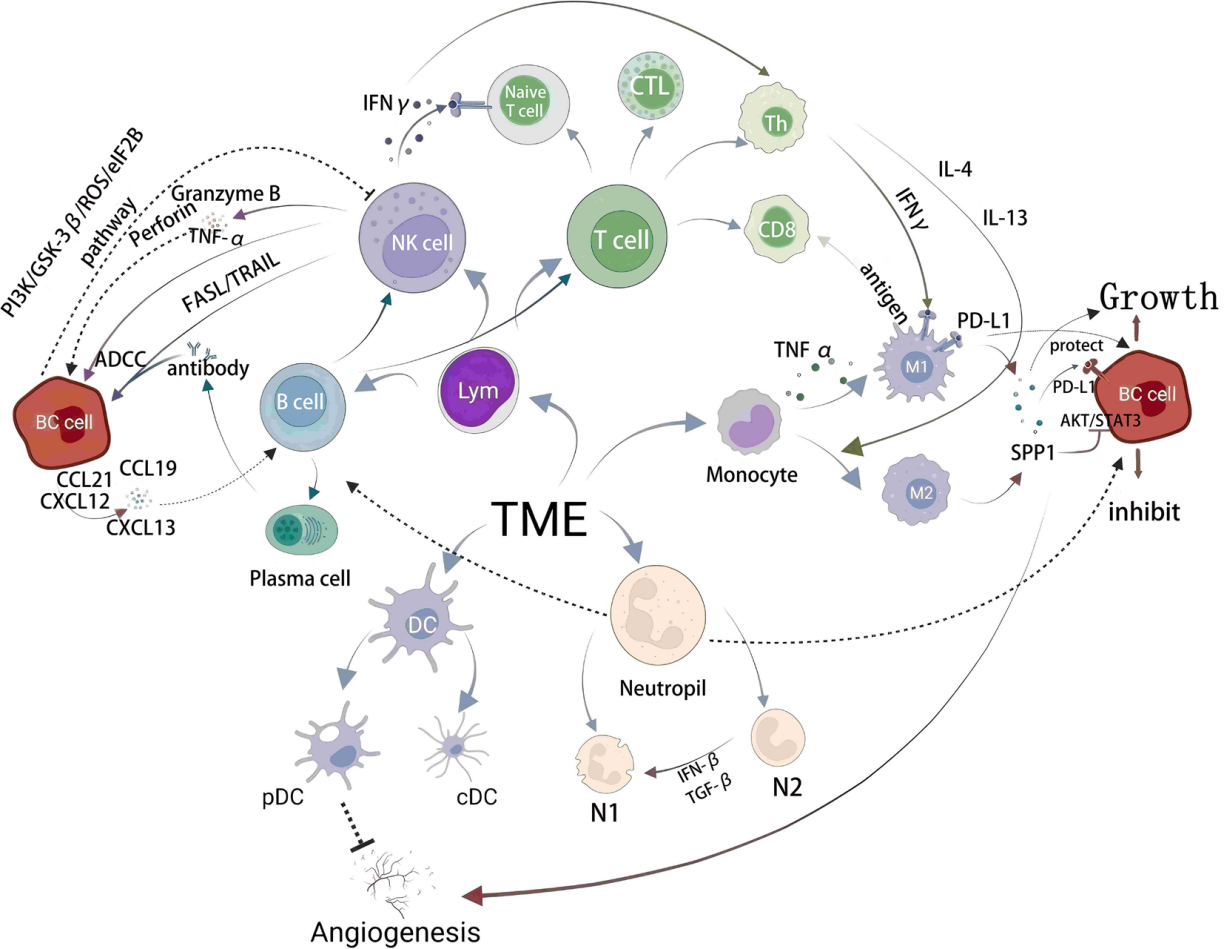
Interactions and regulatory mechanisms of immune cells within the breast cancer tumor microenvironment (TME). This schematic illustrates the diverse immune cell populations and their complex interactions within the BC TME. Tumor‐associated macrophages (M1/M2 TAMs) influence tumor progression by secreting cytokines (e.g., SPP1, PD‐L1), promoting tumor growth and immune evasion. Cytotoxic T lymphocytes (CTLs) and NK cells mediate direct tumor cell killing via granzyme B, perforin, FASL/TRAIL, and cytokines (IFNγ, TNFα). Dendritic cells (DCs), including conventional DCs (cDCs) and plasmacytoid DCs (pDCs), coordinate adaptive immunity through antigen presentation and regulation of angiogenesis. B and plasma cells (PCs) contribute to antitumor responses via antibody‐dependent cell‐mediated cytotoxicity (ADCC). Neutrophils exhibit dual phenotypes (N1 and N2), regulated by cytokines (IFN‐β, TGF‐β), which affect tumor progression and immunosuppression. In addition, monocytes differentiate into polarized macrophages, modulating local inflammation, while chemokines (CCL19, CCL21, CXCL12, CXCL13) recruit various immune cells to shape the immune milieu further. Intrinsic tumor signaling pathways (AKT, STAT3) promote tumor progression and immune evasion by inducing PD‐L1 expression. Overall, these interactions define the immune microenvironment in BC and represent promising therapeutic targets.

#### 3.1.1 TAMs

Macrophages perform critical functions in vivo by engulfing foreign substances, maintaining tissue homeostasis, and secreting hormones and cytokines, thereby participating in inflammatory responses, immune regulation, and tissue repair. Under various microenvironmental stimuli, macrophages differentiate into pro‐inflammatory (M1) or anti‐inflammatory (M2) phenotypes. An imbalance between these two subtypes significantly influences disease progression; consequently, extensive research aims to modulate macrophage numbers and phenotypes for therapeutic purposes [11]. In TME, TAMs primarily promote tumor progression by inducing angiogenesis and remodeling the extracellular matrix (ECM), suppressing anti‐tumor immunity, and facilitating tumor immune evasion and metastasis. Studies have demonstrated that secreted phosphoprotein 1 (SPP1), secreted by TAMs, serves as an intercellular mediator, enhancing the pro‐tumor functions of TAMs, elevating PD‐L1 expression, and sustaining cancer cell stemness. Moreover, the AKT and STAT3 signaling pathways play essential roles in BC cell proliferation, migration, and invasion [12]. Further research indicated that inhibition of SPP1 effectively decreases N‐cadherin and β‐catenin expression, simultaneously blocking AKT and STAT3 activation and significantly reducing tumor cell invasion and metastasis [7].

Recent studies have also highlighted the critical role of APOE^+^ macrophages in tumor immune evasion and poor responses to ICIs. In TNBC, patients with poor responses to ICIs exhibit higher proportions of APOE^+^ macrophages. Multiplex immunohistochemical staining (mIHC) and in vivo studies have confirmed that interactions between APOE^+^ macrophages and CD8^+^ exhausted T cells (Tex) markedly reduce the efficacy of ICIs. In the 4 T1 murine BC model, combined APOE inhibitor and ICI therapy exhibited optimal anti‐tumor effects, suggesting that targeting APOE^+^ macrophages could potentially enhance the therapeutic efficacy of ICIs [13]. Notably, TAMs are the predominant PD‐L1‐expressing cells in human tumors. Studies suggest PD‐L1‐positive TAMs exhibit immunostimulatory properties, while PD‐L1‐negative TAMs suppress T‐cell activation and function, further promoting tumor immune escape [14, 15]. This observation underscores the need to distinguish between PD‐L1‐positive and PD‐L1‐negative TAMs to develop more precise therapeutic strategies.

#### 3.1.2 TILs

In BC, TILs represent the predominant immune cell population, primarily composed of CD8^+^ and CD4^+^ T cells, alongside fewer NK and B cells [16]. The degree of TIL infiltration correlates closely with immune therapy responses and prognosis, serving as a critical predictive indicator [17].

CD8^+^ T cells exert anti‐tumor effects by recognizing antigens presented by Major Histocompatibility Complex (MHC) class I molecules, and higher infiltration typically predicts better outcomes [18]. Concurrently, CD4^+^ T‐cell clonality reflects tumor antigen heterogeneity; memory and mature CD4^+^ T cells associate with favorable prognosis, whereas increased regulatory T cells (Tregs) predict poor outcomes. Cytotoxic CD8^+^ T lymphocytes (CTLs), essential anti‐tumor effector cells, frequently become dysfunctional and exhausted within the immunosuppressive TME [19].

Recent mouse studies indicate circadian rhythms in CD8^+^ T cells correlate with survival and anti‐PD‐1 treatment responses in melanoma patients, highlighting circadian modulation of TME as a promising therapeutic direction [20]. Moreover, CD19^+^LAG‐3^+^ B cells in tumor‐draining lymph nodes (tDLNs) are associated with cell proliferation and potentially favorable outcomes. However, the specific role of B cells in BC, including systemic anti‐cancer responses and metastasis surveillance, remains insufficiently characterized [21, 22]. Overall, coordinated infiltration and activation of B and T cells critically influence metastatic BC lesions, emphasizing their synergistic importance.

#### 3.1.3 NK Cells

NK cells mediate cytotoxicity through granzyme B, perforin release, pro‐inflammatory cytokines, and apoptosis induction via the FASL/TRAIL pathway [23]. Although the TME can suppress NK cell function, enhancing NK cell activity through cytokine supplementation, inhibition of immune‐suppressive factors, or genetic engineering shows significant therapeutic potential against solid and hematologic malignancies.

Thacker et al. reported high Socs3 expression, and CD11b^−^CD27^−^ immature NK cells were associated with poorer TNBC prognosis, potentially due to reduced granzyme‐mediated cytolysis and increased Wnt16 expression promoting tumor metastasis. These findings suggest that immature NK cells are prospective therapeutic targets [24, 25]. Jin et al. showed that BC cells enhance tumor growth and lung metastasis by modulating NK cell activity through the PI3K/GSK‐3β/ROS/eIF2B pathway [26].

Emerging CAR‐NK cell therapies expressing chimeric antigen receptors demonstrate substantial response rates and favorable safety profiles in various cancer clinical trials [27, 28]. Anti‐CD19 CAR‐NK clinical trials for lymphoma currently exhibit a 73% response rate with minimal toxicity, underscoring the promising clinical prospects of NK cell therapy [28].

#### 3.1.4 DCs

DCs, critical for immune surveillance, bridge innate and adaptive immunity [29]. They are functionally divided into conventional (cDCs) and plasmacytoid DCs (pDCs), primarily secreting interferons. Although rare, tumor‐infiltrating pDCs exhibit significant dysfunction, correlating with poor prognosis. pDCs can inhibit angiogenesis pathways, suggesting potential anti‐tumor effects that remain underexplored therapeutically [24]. DCs initiate T‐cell activation and proliferation and regulate effector CD4^+^ T‐cell differentiation, significantly influencing immune status within the TME [30, 31]. However, impaired DC function and inadequate immune activation remain significant barriers. DC vaccines, designed to activate lymphocytes and continuously surveil tumors, have demonstrated preclinical potential but currently face challenges of limited specificity and effective biomarkers [31, 32]. Future research should enhance DC vaccine efficacy, possibly through combinations with ICIs or adoptive cell therapies [33].

#### 3.1.5 Tumor‐Associated Neutrophils

Neutrophils exhibit either anti‐tumor (“N1”) or pro‐tumor (“N2”) phenotypes regulated by IFN‐β and TGF‐β signaling [34]. Blocking TGF‐β enhances neutrophil anti‐tumor activity and CD8^+^ T‐cell activation. Low‐density neutrophils in BC are associated with myeloid‐derived suppressor cells (MDSCs) and TGF‐β‐mediated immunosuppression, further advancing tumor progression [35]. Additionally, neutrophil extracellular traps (NETs) facilitate immune escape and treatment resistance by secreting immunosuppressive factors and recruiting Tregs. Although complex interactions exist between tumor‐associated neutrophils (TANs) and other infiltrating immune cells, the specific mechanisms require further clarification [36, 37].

### 3.2 Roles and Advances of Other Immune Cells

In addition to the primary immune cells, plasma cells (PCs), mast cells, monocytes, eosinophils, and basophils contribute to anti‐tumor immunity [38].

#### 3.2.1 PCs

The precise role of plasma cells in local tumor immunity remains unclear. However, they mediate antibody‐dependent cellular cytotoxicity (ADCC) by producing tumor‐specific IgG1 antibodies, which bind Fc gamma receptors (FcγR), promoting tumor cell clearance and positively correlating with patient prognosis [39]. Further investigation into plasma cell functions in BC immunity could inform new antibody‐based therapeutic strategies.

#### 3.2.2 Cancer‐Associated Fibroblasts

Derived from the dedifferentiation of local fibroblasts, cancer‐associated fibroblasts (CAFs) regulate T‐cell function and immune tolerance by upregulating immune checkpoint molecules. By remodeling the ECM through biglycan, CAFs suppress the activity of NK and CD8^+^ T cells and activate immunosuppressive macrophages. However, due to subtype diversity and limited research, therapeutic targeting of CAFs remains challenging [40].

#### 3.2.3 Mast Cells

Mast cells are distinct tissue‐resident immune cells capable of secreting various bioactive substances that stimulate, modulate, or suppress immune responses. Increasing evidence highlights mast cell infiltration in BC; however, whether they promote or inhibit BC progression remains controversial. Further studies are required to elucidate their mechanisms and roles in anti‐tumor immunity.

#### 3.2.4 Other Innate Immune Cells

Unconventional T cells, including γδ T cells, NK T cells, and mucosal‐associated invariant T cells, have gained attention for their potential regulatory roles. These cells, characterized by semi‐invariant T‐cell receptors, regulate and mediate anti‐tumor responses, making them potential targets for immunotherapy [41].

### 3.3 Emerging Mechanisms of Immune Cells

**FIGURE 4:**
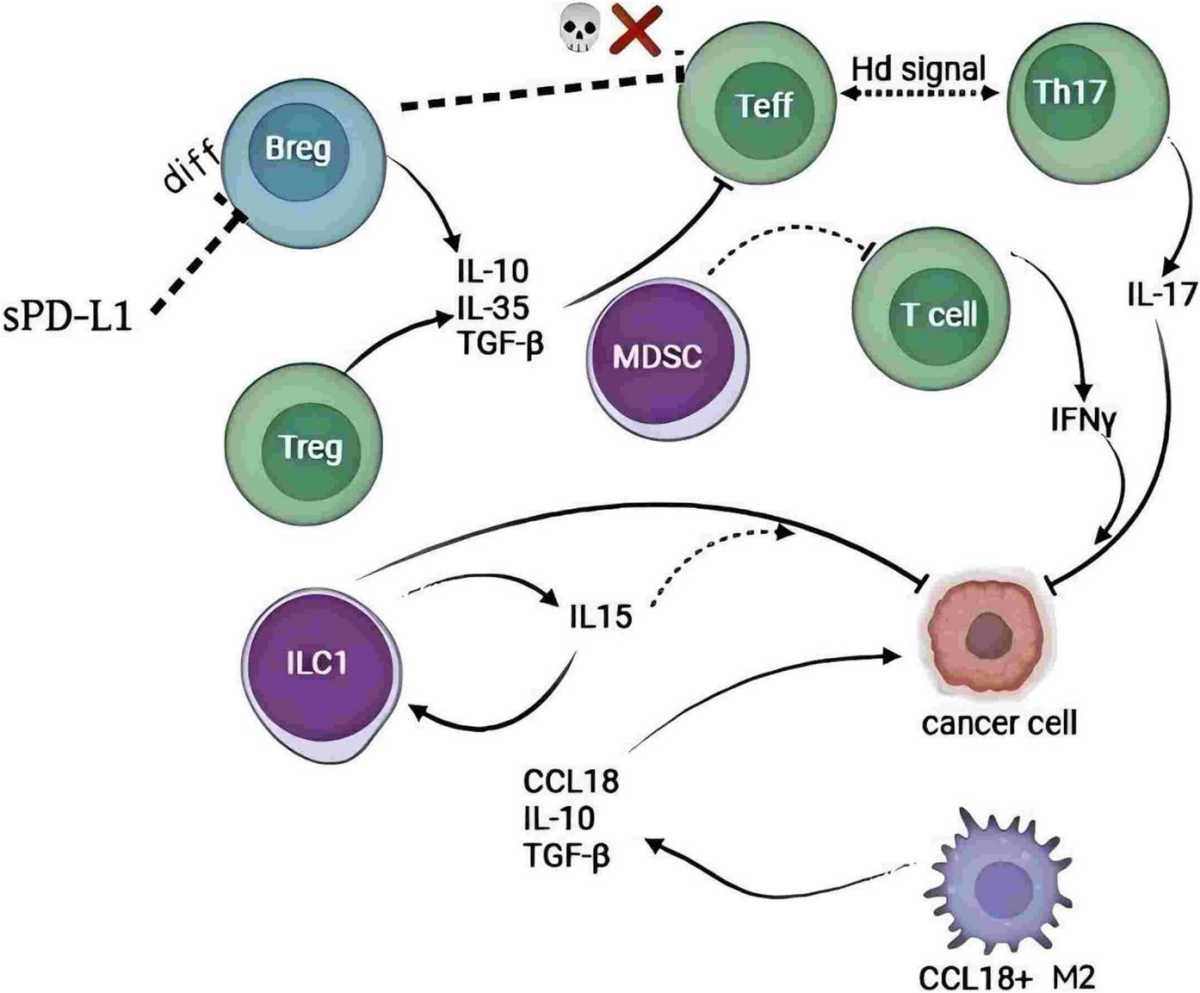
Schematic diagram of emerging immune cell subpopulations and their interactions in the breast cancer immune microenvironment. This figure highlights the principal immune cell subsets within the BC microenvironment and their key secreted factors, including Bregs, Tregs, MDSCs, Th17 cells, effector T cells (Teff), innate lymphoid cells (ILC1), and CCL18^+^ M2 macrophages. Solid and dashed arrows denote direct or indirect regulatory relationships among these cells; annotations indicate secreted cytokines (e.g., IL‐10, IL‐35, TGF‐β, IL‐17, IFNγ, IL15, CCL18) and key signaling pathways (e.g., Hedgehog/Hd signal). The interplay among these immune cells establishes an immunosuppressive microenvironment that promotes tumor growth and metastasis while offering potential targets for tailored immunotherapy.

Recent studies have unveiled a pronounced immunosuppressive microenvironment within metastatic BC lesions, pivotal in tumor immune evasion and metastatic progression. Figure 4 systematically illustrates the intricate interactions among various immune cells—such as CCL18^+^ M2 macrophages, MDSCs, regulatory B cells (Bregs), Tregs, innate lymphoid cells (ILC1), T helper 17 cells (Th17), and effector T cells (Teff)—and their associated cytokines, including IL‐10, IL‐35, TGF‐β, IL‐17, and IFNγ. The regulatory network formed by these cells and factors is crucial in the immunosuppression and progression of BC. Below are some emerging immune cell mechanisms and their related findings.

#### 3.3.1 Emerging Macrophage Subpopulations (CCL18 ^+^ M2 Macrophages)

High‐resolution mapping of the tumor ecosystems in BC liver and brain metastases, achieved through single‐cell RNA sequencing, has enriched CCL18^+^ M2 macrophages. This subpopulation is closely associated with tumor immune evasion and metastatic progression, offering new avenues for optimizing immunotherapeutic strategies in BC [42].

#### 3.3.2 Innate Lymphoid Cells

Innate lymphoid cells (ILCs) represent a recently identified subset of lymphocytes lacking typical T and B cell receptors and are classified into ILC1, ILC2, and ILC3 [43]. Studies indicate that ILC1 enhances anti‐tumor responses in murine BC models by interacting with cancer cells and producing IL‐15 and IFNγ, thereby establishing an immune surveillance mechanism against epithelial malignancies [44]; conversely, the infiltration of ILC3 is correlated with BC progression, suggesting that future research may focus on modulating ILC activity to improve immune surveillance and therapeutic outcomes.

#### 3.3.3 MDSCs

MDSCs promote tumor immune evasion primarily by secreting factors such as IL‐10 and TGF‐β and interacting with T cells and other immune cells, posing a significant challenge in cancer immunotherapy. The development of carrier‐free nanoparticles, like gemcitabine‐celecoxib nanoparticles (GEM‐CXB NP), provides novel approaches for chemoimmunotherapy in BC [45]. Therefore, therapeutic strategies targeting MDSC signaling pathways remain a focal point for future research.

#### 3.3.4 Bregs and T Cells (Tregs)

Bregs contribute to tumor immune evasion by secreting IL‐10, IL‐35, and TGF‐β, which inhibit T‐cell activity and promote immune tolerance. In BC, elevated levels of soluble PD‐L1 (sPD‐L1) enhance Breg proliferation and IL‐10 secretion, facilitating Treg differentiation and potentially leading to the exhaustion of Teff function, indicating that targeting Bregs may enhance anti‐tumor immunity [46]. On the other hand, Tregs maintain immune balance by secreting anti‐inflammatory factors and promoting M2 macrophage polarization; however, their hyperactivity can undermine anti‐tumor immunity. Studies have found that the key transcriptional cofactor SRC‐3 is essential for maintaining immune homeostasis in Tregs; disrupting SRC‐3 specifically in Tregs not only aids in tumor eradication but also protects healthy tissues from inflammation [47].

#### 3.3.5 T Helper 17 Cells

T helper 17 cells (Th17) cells participate in BC immune evasion by secreting IL‐17. Research indicates that neutralizing IL‐17 and IFNγ can inhibit the migration of BC cell lines (BCCLs), demonstrating their pathogenic roles [48]; conversely, supplementing IL‐17 enhances BCCL migration, with IFNγ further amplifying this effect. Additionally, obesity‐related mesenchymal stem cells have been found to induce inflammation and increase immune checkpoint inhibitory markers by activating pathogenic Th17 cells, thereby accelerating BC invasion [49]. Moreover, the Hedgehog (Hh) signaling pathway plays a significant role in the reciprocal regulation between Teff and Th17 cells, with its aberrant activation potentially promoting BC immune evasion and progression [50].

#### 3.3.6 Drug Resistance and miR‐200c

In studies on drug resistance in BC, the restorative expression of miR‐200c has been shown to increase the sensitivity of BC cells to the chemotherapeutic agent cytarabine. This finding suggests that miR‐200c is critical in BC drug resistance and may serve as a potential therapeutic target to enhance chemotherapy efficacy [51].

## 4 Fcγ Receptors and Their Genetic Polymorphisms

Fcγ receptors (FcγRs) are expressed on the surface of immune cells and can recognize and bind antibodies on tumor cells, mediating innate and adaptive immune responses. Their activation is influenced by genetic polymorphisms, which can alter antibody‐effector cell interactions, modulating the binding affinity of IgG Fc and the efficiency of antibody‐dependent cellular cytotoxicity (ADCC) [52]. Furthermore, Fc glycosylation affects FcγR binding and ADCC. Current research explores the role of FcγRIIIa polymorphisms in other cancer immunotherapies and their potential to enhance antibody treatment efficacy [39, 53].

The heterogeneity of immune cells and immunosuppressive mechanisms in BC underscores the complexity of tumor immune evasion and progression. By modulating the functions of MDSCs, Bregs, Tregs, and Th17 cells, new strategies for BC immunotherapy may emerge. Future research will focus on exploring more efficient immunomodulatory approaches to optimize BC treatment outcomes and overcome current challenges in immunotherapy.

## 5 Discussion

**FIGURE 5:**
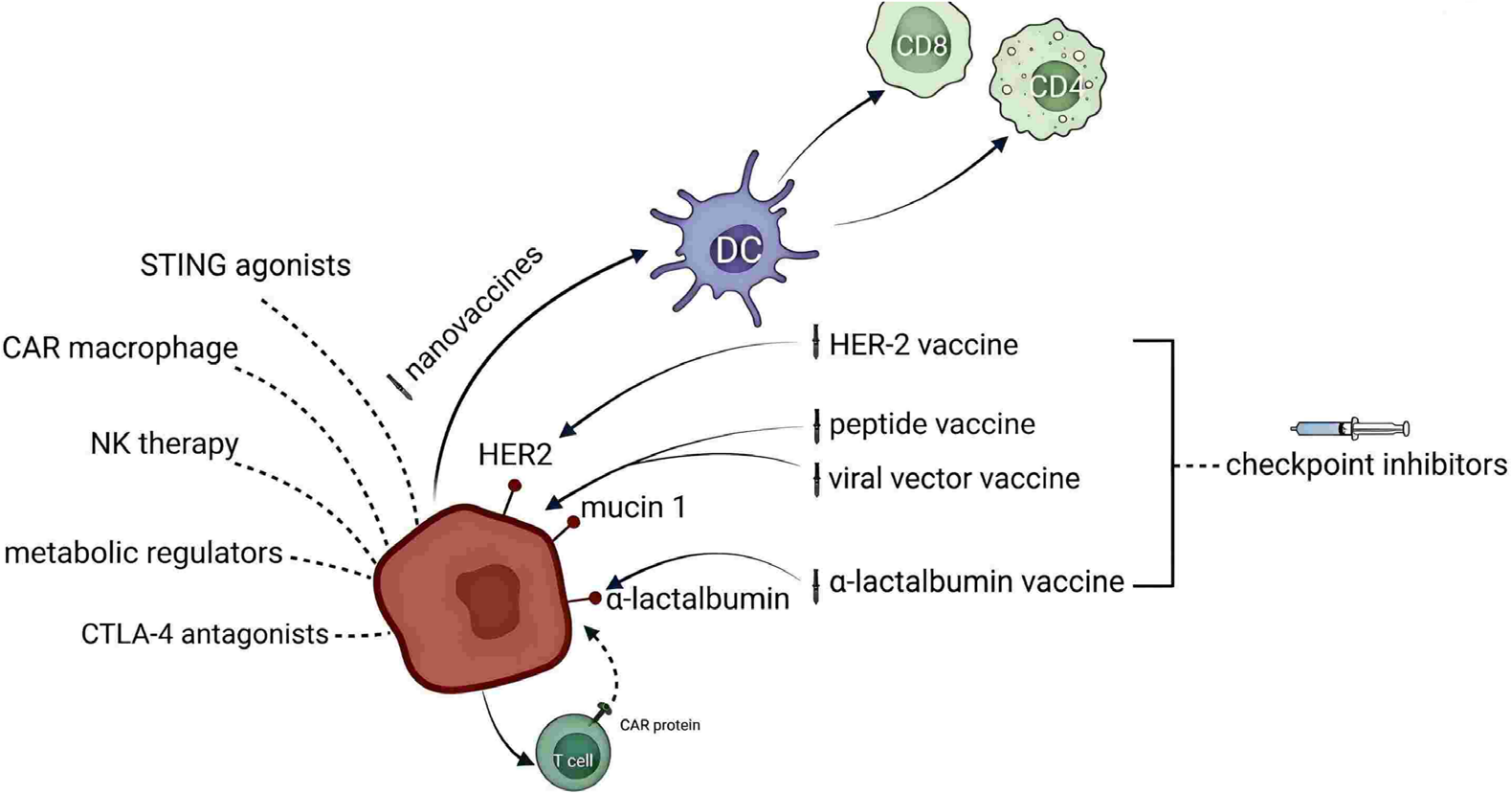
Schematic of breast cancer immunotherapy strategies and their mechanisms of interaction. This figure depicts the interplay between BC cells (red) and common tumor‐associated antigens (HER‐2, mucin 1, αlactalbumin) with various emerging immunotherapeutic strategies. Dendritic cells (DCs) act as a bridge by presenting antigens and activating CD4^+^ and CD8^+^ T cells to initiate and enhance the adaptive immune response. Additionally, several vaccine strategies (including HER2, peptide, viral vector, α‐lactalbumin, and nano vaccines) directly target tumor antigens to exert antitumor effects, while combination with immune checkpoint inhibitors further amplifies the immune response. Other emerging immunomodulatory approaches—such as STING agonists, CAR‐macrophages, NK cell therapy, metabolic regulators, CTLA‐4 antagonists, and CAR‐T cell therapy—enhance therapeutic outcomes by boosting innate immunity, improving the tumor microenvironment, or directly targeting tumor cells. Integrating these diverse immunotherapeutic strategies collectively provides robust support and a theoretical basis for improving clinical outcomes in BC.

Figure 5 illustrates the interactions between BC cells (depicted as red cells) expressing common antigens such as HER‐2, mucin 1, and α‐lactalbumin and various immunotherapeutic strategies. It also highlights the pivotal roles of DCs, NK cells, and other immune regulatory molecules in the anti‐tumor response. This schematic depicts how different immunotherapeutic approaches—including vaccines, chimeric antigen receptor T (CAR‐T) cells, CTLA‐4 inhibitors, and STING agonists—can synergistically modulate TME and enhance therapeutic efficacy.

### 5.1 Current Clinical Applications

TNBC is often characterized by pronounced fibrosis and ECM deposition within the TME, which not only hampers drug delivery but also exacerbates tumor immune evasion. Studies have demonstrated that alleviating hypoxia, optimizing vascular structures, and modulating tumor‐associated immune cells can partially reverse this immunosuppressive state, thereby enhancing treatment efficacy [54]. For instance, the PD‐L1 inhibitor atezolizumab has shown durable clinical benefits in patients with metastatic TNBC, underscoring the potential of TME modulation [54]. Additionally, the combination of adagloxad simoleon and OBI‐821 has exhibited immune activation effects in early‐stage Globo‐H–positive TNBC patients, although long‐term efficacy remains to be further validated [55].

Despite the FDA's approval of anti‐PD‐1/PD‐L1 ICIs for TNBC treatment, challenges such as low response rates and the absence of predictive biomarkers persist [8, 56, 57]. Moreover, while CAR‐T cell therapy has succeeded in hematologic malignancies, its application in solid tumors like BC is significantly limited by the complex immunosuppressive TME. BC‐on‐a‐chip models can assess CAR‐T cell infiltration and efficacy but fail to replicate the intricate clinical TME [58] entirely. The molecular heterogeneity of TNBC further constrains the development of universal targeted therapies, suggesting that personalized treatment strategies, such as combining genetic profiling with immunotherapy, will be pivotal for future precision medicine [59]. Recent studies silencing both VISTA and CTLA‐4 have provided new avenues to enhance T‐cell anti‐tumor responses, potentially overcoming the issue of low immunotherapy response rates [60]. Additionally, research indicates that the STING agonist ADU‐S100, in combination with the PD‐1 blocker natalizumab, is well‐tolerated and exhibits clinical activity in TNBC patients, further confirming the potential of combination immunotherapies [61].

While clinical immunotherapy has made notable strides, issues such as low response rates, TME heterogeneity, and the lack of effective biomarkers remain prominent, necessitating personalized and combination treatment strategies to improve therapeutic outcomes.

### 5.2 Vaccines and Immune Response

BC vaccines primarily aim to treat existing tumors by activating specific immune responses and establishing long‐lasting immune memory, thereby eliminating residual cancer cells and achieving sustained tumor control [62, 63]. Current vaccine research, including DC and specific antigen vaccines, is based on this concept. For example, vaccines utilizing zinc phosphate nanoparticles to promote DC maturation have demonstrated significant anti‐tumor potential in animal models [64, 65]. Similarly, α‐lactalbumin vaccines, which induce autoimmune degradation of breast tissue, have shown promising results in prevention and early treatment and are currently undergoing phase I clinical trials to evaluate safety and optimal dosing [54, 63]. However, these vaccines' clinical efficacy and safety require further long‐term investigation.

BC vaccines' ability to activate immune responses and establish immune memory offers a novel strategy for tumor control. Nevertheless, their long‐term efficacy, safety, and synergy with other immunotherapies need validation in larger‐scale clinical trials.

### 5.3 Limitations of Immunotherapy and Combination Treatment Strategies

While advantageous, single‐agent immunotherapies, such as vaccines or ICIs, often struggle to overcome TME heterogeneity, immune evasion, and drug resistance challenges. Currently, various combination treatment approaches are under exploration.

#### 5.3.1 Vaccine and ICIs Combination Therapy

Enhancing T‐cell activity while reducing immune suppression theoretically improves overall treatment efficacy [66]. Although ICIs alone can provide durable anti‐tumor responses, their effectiveness is limited by tumor heterogeneity and TME immunosuppression. Multiple combination immunotherapy regimens are in clinical trial phases, necessitating further clarification of optimal combinations and personalized treatment strategies [67, 68, 69].

#### 5.3.2 Targeting Immunosuppressive Cells

Studies have identified elevated APOE^+^ macrophages in patients unresponsive to ICI therapy. Preliminary animal research suggests that targeting this subset can enhance ICI efficacy, warranting further clinical validation of this combination strategy [13].

#### 5.3.3 Dual Immune Checkpoint Inhibition

TME heterogeneity, particularly in patients with brain metastases, significantly impedes BC immunotherapy effectiveness [8]. Recent research demonstrates that dual silencing of VISTA and CTLA‐4 markedly enhances anti‐tumor immune responses, improves the inflammatory cytokine milieu, and inhibits tumor progression, offering a novel approach to address TME heterogeneity [60].

#### 5.3.4 The Optimization of CAR‐T Cell Therapy

CAR‐T cell therapy has progressed in Luminal A‐type BC, but challenges remain in Luminal B‐type BC. Future efforts should optimize CAR constructs and explore combination therapies to overcome persistent resistance, immune evasion, and toxicity in solid tumor treatments [70, 71].

Additionally, genetically engineered CAR macrophages capable of specifically recognizing and killing BC cells present a promising strategy to overcome tumor immune evasion. Metabolic modulators targeting TME pathways may enhance immunotherapy efficacy by improving immune cell function or inhibiting tumor metabolism.

The interactions among various cells depicted in Figure 5—such as tumor cells, DCs, and NK cells—and their corresponding immunotherapeutic strategies not only enhance our understanding of treatment mechanisms but also underscore the potential of combination therapies in modulating TME and improving therapeutic outcomes. This insight provides a theoretical foundation for developing personalized treatment strategies in the future.

Overall, combination therapy strategies hold promise in addressing the limitations of monotherapies. However, optimizing the synergy among different treatment modalities, determining the most effective combinations, and achieving individualized treatment remain critical areas for future research.

## 6 Conclusion

Recent advances in immunotherapy have introduced novel approaches for treating refractory solid tumors such as BC, significantly transforming cancer treatment paradigms. However, our review has identified several key knowledge gaps, particularly regarding tumor immune evasion mechanisms, TME heterogeneity, and the complex interactions among various immune cell subpopulations. While pivotal roles of immune cells in BC initiation, progression, and treatment have been demonstrated, challenges such as low response rates, the absence of robust predictive biomarkers, and the limited efficacy of monotherapies persist. These findings underscore the need for a deeper mechanistic understanding and the development of innovative strategies to overcome TME‐associated immunosuppression. Innovative therapies based on gene editing, novel immune cell carriers, and the combined application of ICIs, CAR‐T cell therapies, and innate immune strategies hold promise for breakthroughs in BC treatment. Future research should prioritize elucidating the molecular underpinnings of immune regulation, optimizing combination treatment protocols, and validating predictive biomarkers to facilitate the clinical translation of personalized immunotherapy approaches, ultimately improving patient outcomes and QoL.

## Author Contributions

**R.L.:** conceptualization (lead); writing – original draft (lead); writing – review and editing (equal). **N.C.:** supervision (lead); conceptualization (supporting). **W.L.:** project administration (supporting); writing – review and editing (supporting). **D.L.W.:** data curation (supporting); writing – review and editing (supporting). All authors: final approval of the manuscript (equal).

## Disclosure

All cell illustrations were created using BioRender.com.

## Ethics Statement

The authors have nothing to report.

## Conflicts of Interest

The authors declare no conflicts of interest.

## Supporting information


**Data S1.** Supporting Information.

## Data Availability

The data that support the findings of this study are available from the corresponding author upon reasonable request.
